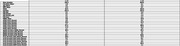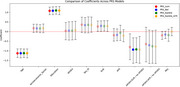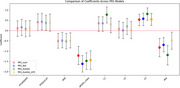# Application of a Polygenic Risk Score (PRS) Composed of Five PRSs in Admixed Brazilian and European Populations

**DOI:** 10.1002/alz70860_107188

**Published:** 2025-12-23

**Authors:** Samantha LG Paço, Shea J Andrews, Michel Satya Naslavsky

**Affiliations:** ^1^ Universidade de São Paulo, São Paulo, Sao Paulo, Brazil; ^2^ Department of Psychiatry and Behavioral Sciences, University of California ‐ San Francisco, San Francisco, CA, USA; ^3^ University of São Paulo, São Paulo, Brazil

## Abstract

**Background:**

Alzheimer's disease (AD) is linked to cognitive decline, with genetic factors like the APOE4 allele increasing risk. Polygenic risk scores (PRS) combine genetic variants to predict AD susceptibility. This project applies PRS models to two datasets: SABE (Brazilian admixed elderly population) and ADNI (European population), aiming to understand how genetic factors and ancestry influence cognitive impairment.

**Method:**

PRS are generated using PRSice‐2, including Sofer et al.'s PRS_SUM for cognitive impairment prediction. Models analyzed: Bel (1,937 SNPs), Kunkle (12,002 SNPs), Kunkle‐AFR (157 SNPs), and PRS_SUM (14,107 SNPs). The Dataset areSABE ‐ ABraOM: 1,103 Brazilian elderly individuals, 64% European ancestry. ADNI: 785 individuals, mostly European ancestry, with clinical data on AD.

**Statistics**: Linear regression with bootstrapping (*n* = 1000), considering variables like age, sex, education, APOE4, and local ancestry.

**Results:**

In SABE, age negatively impacts cognition, while education improves it. APOE4's effect is small but suggests higher risk in individuals with European ancestry. PRS models vary, with *R* ^2^ between 0.266 and 0.268. Local ancestry analysis shows greater homozygosity in European and East Asian ancestries.

In ADNI, APOE4 significantly affects MMSE, with age also negatively impacting cognition. PRS_Kunkle explained more variability (*R* ^2^=0.155), while PRS_Kunkle_AFR explained less (*R* ^2^=0.131).

**Conclusion:**

The project highlights the complex relationships between PRS, cognitive performance, and genetic ancestry in AD. PRS models vary across populations, and local ancestry analysis in SABE emphasizes the importance of considering ancestry in admixed populations.